# A suggested method for setting up GSI profiles on the GE Revolution CT scanner

**DOI:** 10.1002/acm2.12754

**Published:** 2019-11-05

**Authors:** David M. Gauntt

**Affiliations:** ^1^ Department of Radiology University of Alabama at Birmingham Medical Center Birmingham AL USA

**Keywords:** computed tomography, CT AEC, CT protocols, dual energy CT, GSI assist

## Abstract

“GSI Assist” is the automatic exposure control (AEC) system for dual‐energy acquisitions on the GE Revolution CT scanner. This paper describes the user options of GSI Assist, and describes the method developed at UAB Medical Center to simplify the use of GSI Assist without adversely affecting the AEC Operation.

## 1 Introduction

CT automatic exposure control (AEC) is commonly used for chest, abdomen, and pelvis CT exams. AEC systems adjust the tube current (and on some systems the tube voltage^1^, ^2^, ^3^) to accommodate differences in patient size, both from patient to patient and within a single patient acquisition, in order to simplify the task of obtaining adequate image quality without the use of excessive radiation dose.^4^, ^5^

Dual‐energy scanners acquire two independent sets of attenuation data with different energy‐dependent characteristics. Dual energy General Electric Revolution CT scanners accomplish this by changing the tube voltage from one x‐ray pulse to the next at a rate of more than 1000 pulses per second.^6^ The tube current may vary from exam to exam, but the high‐energy and low‐energy tube currents are each fixed during a single exam, and the tube voltages do not vary from exam to exam.

### 1.1 Overview of CT automatic exposure control

Most modern scanners have AEC systems. These systems use localizer views to determine the patient size, and then adjust the tube current accordingly, using higher currents for larger patients and lower currents for smaller patients. AEC systems usually also have tube current modulation options.^7^ Longitudinal modulation (or z‐modulation) adjusts the tube current as the patient thickness changes along the length of the patient. Angular modulation (or rotational modulation) uses higher tube currents for lateral views and lower tube currents for anterior/posterior views; this allows for a reduction in noise without increasing patient dose. A variation on angular modulation uses a lower tube current when the tube is anterior to the patient, and a higher tube current when posterior. This lowers patient dose to the front of the body, where most radiosensitive organs are located, without increasing image noise.^8^

### 1.2 Automatic exposure control in GE Revolution scanners

#### 1.2.1 Single voltage mode

On the Revolution scanner, single voltage AEC is called “SmartMA.” In single‐voltage scans, the user chooses a setting called the “noise index,” which is part of the imaging protocol but which can be overridden by the technologist at scan time. The AEC system uses the localizer images (called “scout” images on GE scanners) to determine the patient thickness, calculates the CTDI_vol_ required to produce primary patient images whose noise is equal to the noise index, and then calculates the tube current required to produce this CTDI_vol_. Since the noise is also affected by the primary image thickness and iterative reconstruction (ASIR‐V) settings of the primary reconstruction series, these settings also affect the tube current.^6^ However, the choice of filter kernel affects image noise but does not affect the tube current; if the primary series has a kernel other than “Standard,” the noise in the primary series will not match the noise index.

This is similar to other GE scanners, except that in the Discovery and Optima series the AEC takes the primary image thickness into account but not the ASIR setting, and the Optima series scanners have an additional setting called “Dose Reduction” which affects the dose.

#### 1.2.2 Dual energy mode

The dual energy imaging technology used on the Revolution is called "Gemstone Spectral Imaging" (or "GSI"), and dual energy AEC on the Revolution is called “GSI Assist.”  The user selects the “target noise index” (or “target NI”), and the AEC system calculates the CTDI_vol_ (the “target CTDI_vol_”) that would be used for a 120 kV single voltage acquisition without longitudinal modulation when the noise index is set equal to the target NI. The system then selects a combination of high‐voltage and low‐voltage tube currents that would produce a CTDI_vol_ close to the target CTDI_vol_. According to the GSI Xtream Technology White Paper,^6^ the noise in the 70 keV monochromatic image matches the image noise in a dose‐matched single‐energy acquisition. Therefore, the noise in the 70 keV image should be close to the target noise index.

The low‐energy tube current and the high‐energy tube current each remain fixed throughout the acquisition; that is, neither longitudinal nor angular modulation is used. Because longitudinal modulation is not used, the system calculates a single target CTDI_vol_ based on the patient thickness along the entire scan length.

This paper addresses the settings available to the user when setting up protocols on the Revolution, those that are available to the technologist at scan time, and the approach used at the UAB Medical Center to simplify the task of the technologist without adversely affecting image quality or radiation dose.

### 1.3 Details

**Figure 1:**
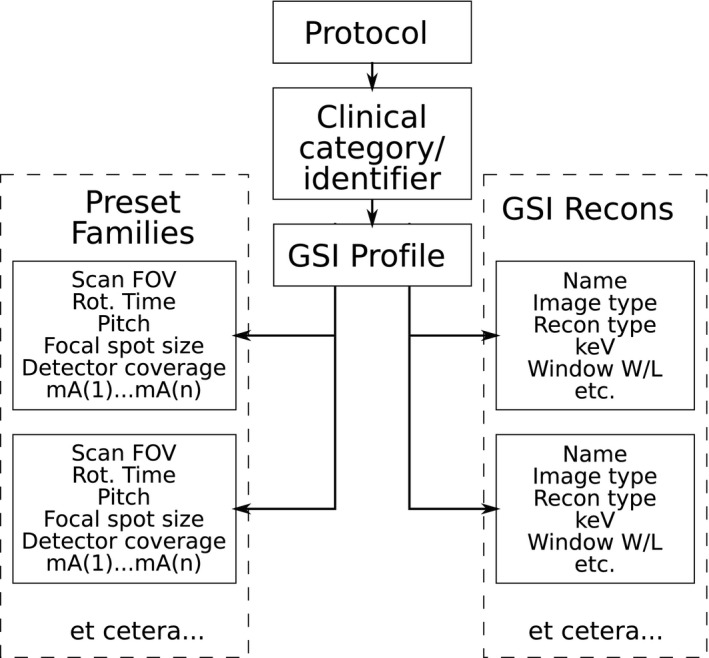
Schematic illustration of hierarchy of GSI settings.

The GSI Assist parameters are arranged in a hierarchy of groups; these groups are the “GSI Preset Family,” the “GSI Recon,” and the “GSI Profile”; this hierarchy is shown in Fig. 1. These groups are described in detail in Appendix C, but here we present an overview of each and how they are related.

- Each Preset Family specifies the gantry rotation time, helical pitch, collimation (i.e. detector coverage), focal spot size, and scan field of view, and includes a list of available tube currents. The user is not able to define new Preset Families. Since each Preset Family allows the use of a range of tube currents, there is a range of values of CTDI_vol_ that can be produced using the particular Preset Family. Appendix B lists the different Preset Families available on the 160 mm version of the Revolution, and the minimum and maximum values of CTDIvol associated with each Preset Family.
- Each GSI Recon specifies reconstruction parameters for one image series. Several GSI Recons are preloaded onto the scanner, but the user can define new GSI Recons. The details of the GSI Recons are listed in Appendix C, but otherwise are beyond the scope of this paper.
- Each GSI Profile specifies a list of Preset Families, as well as a list of GSI Recons. The first Preset Family in the list is the Primary Preset Family, and is always used unless the technologist selects a different Preset Family. Several GE GSI Profiles are preloaded onto each scanner, but the user can define new GSI Profiles.
- A GSI Protocol is simply a CT protocol that includes one or more GSI acquisitions. Each GSI Protocol must specify a Clinical Identifier, which determines which GSI Profile to use. According to GE Medical Systems, in future releases of the software, it will be possible to specify the GSI Profile for each protocol independently of the clinical identifier.

#### 1.3.1 Use of GSI Assist

When a scout view is taken as part of a clinical scan, the scanner will calculate the value of CTDI_vol_ that would be produced by a 120 kV single‐energy acquisition that uses the target noise index. For each Preset Family in the GSI Profile, the scanner determines which of the available tube currents will produce the value of CTDI_vol_ closest to the target CTDI_vol_. If the technologist takes no action, the primary Preset Family will be used even if use of another Preset Family would produce a CTDI_vol_ closer to the target CTDI_vol_. Increasing the number of Preset Families in a GSI Profile can allow the technologist to match the target CTDI_vol_ more closely, or can increase the range of values of CTDI_vol_ available to the technologist.

**Figure 2:**
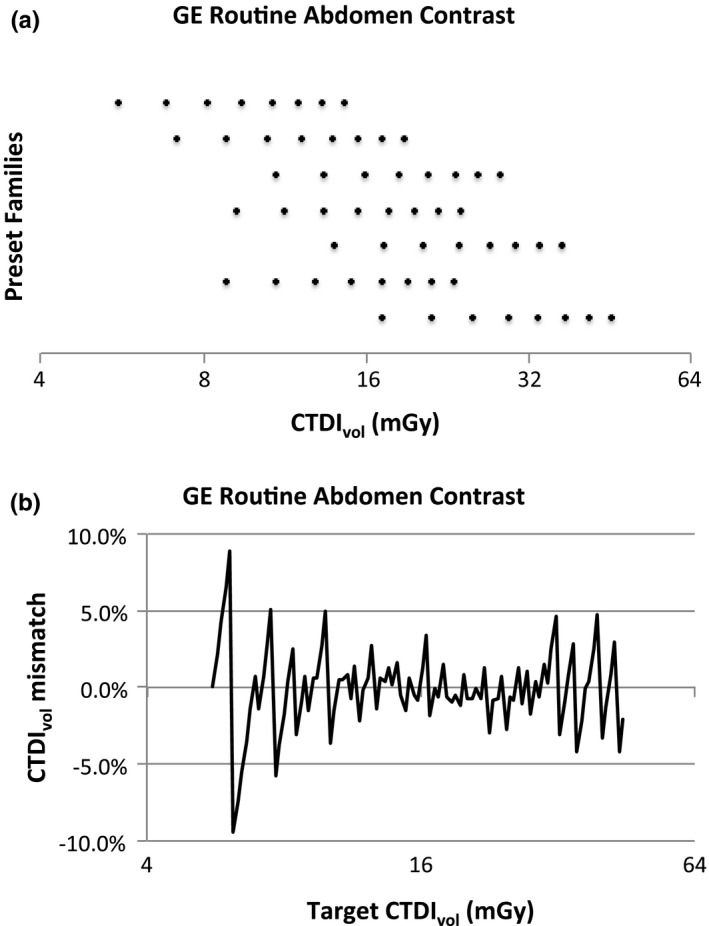
Illustration of the “GE Routine Abdomen Contrast” Reference profile. (a) The values of CTDI_vol_ that can be produced by each Preset Family in the profile. (b) Illustration of the precision with which the system can match any target CTDI_vol_ when using this profile.

**Figure 3:**
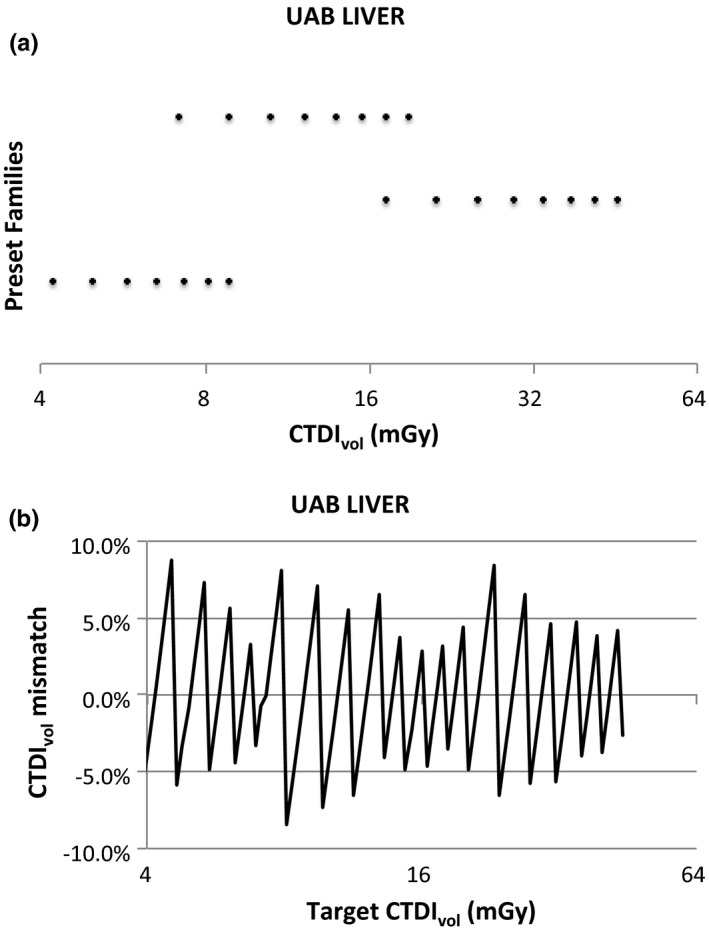
Illustration of the “UAB LIVER” User Profile. (a) The values of CTDI_vol_ that can be produced by each of the three Preset Families in this profile. (b) Illustration of the precision with which the system can match any target CTDI_vol_ when using this profile.

This effect is illustrated in Figs. 2 and 3; these illustrate one Reference Profile (GE Routine Abdomen Contrast) and one User Profile (UAB LIVER). Chart (a) in each figure shows the Preset Families and the values of CTDI_vol_ each can produce. Each row represents the values of CTDI_vol_ that can be produced using a single Preset Family; the top row is the primary Preset Family. Thus, the only values of CTDI_vol_ that can be produced using this GSI Profile are the discrete values represented in chart (a). Chart (b) presents the fractional difference between any target CTDI_vol_ and the closest value of CTDI_vol_ that can be produced using this GSI Profile, calculated as

$$f\left({\text{CTDI}}_{\text{vol}}^{\text{target}}\right)=\frac{{\text{CTDI}}_{\text{vol}}^{\text{target}}-{\text{CTDI}}_{\text{vol}}^{\text{actual}}}{{\text{CTDI}}_{\text{vol}}^{\text{target}}}$$

This is a measure of how closely these values can be matched to a target CTDI_vol_.

The GE Reference Profiles were designed to give the technologist maximum flexibility in selecting the rotation time and helical pitch within certain constraints imposed by clinical considerations. Because the “GE Routine Abdomen Contrast” profile includes many more Preset Families than the “UAB LIVER” profile, there are many more combinations of tube current, rotation time, and helical pitch available, and therefore the target CTDI_vol_ can generally be more closely matched by the technologist. On the other hand, because the “UAB LIVER” profile includes a Preset Family that can produce CTDI_vol_ values down to 4 mGy, a wider range of target CTDIs can be matched than with the reference profile. In addition, the reduced number of options available to the technologist using the “UAB LIVER” profile simplifies the task of selecting the Preset Family.

#### 1.3.2 User interface

**Figure 4:**
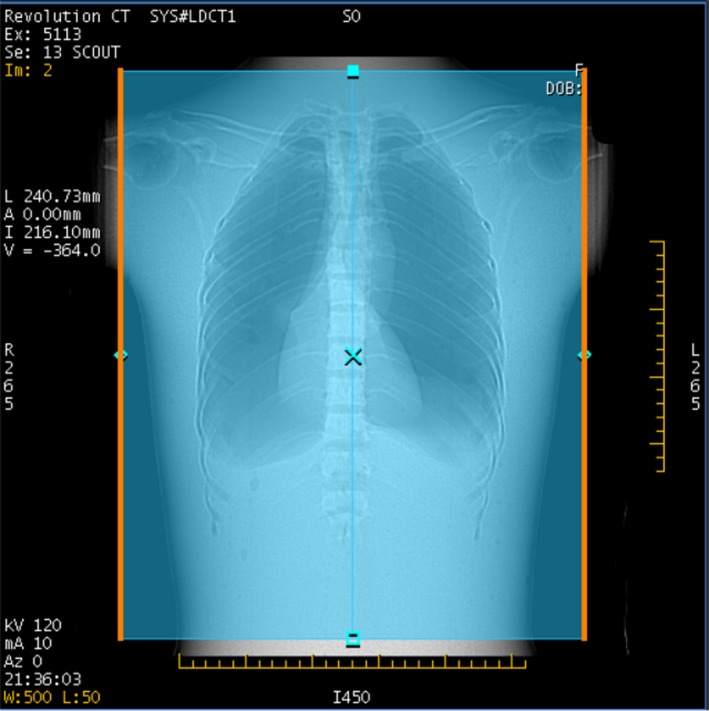
Scout view for GSI scan of chest phantom. The orange lines indicate the anatomy for which the predicted image noise will be above the prescribed noise index.

**Figure 5:**
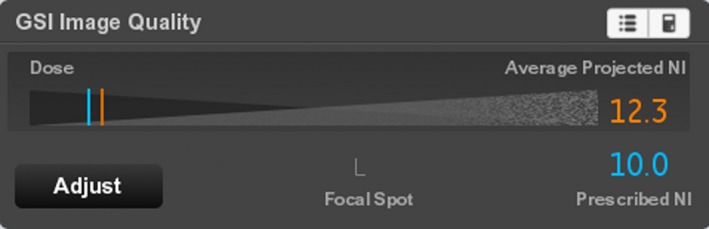
The GSI IQ widget for the scan of Fig. 4. The widget shows the expected image noise in a 70 keV image (“Average Projected NI”) and the target noise index (“Noise index”). The Average Projected NI is displayed in orange when it is higher than the Prescribed NI. The technologist can click on the “Adjust” button to change the selected Preset Family, and thus the Average Project Noise Index.

After the scout view is taken, the scanner displays the scout image (Fig. 4) and the GSI Image Quality widget dialog (Fig. 5). The IQ widget displays both the target noise index, and the “average projected noise index” (AvgPNI), which is an estimate of the image noise averaged over the scan length if the presently selected Preset Family is used. When the IQ widget first appears, the AvgPNI for the primary Preset Family is displayed.

The parts of the anatomy for which the projected noise is above the Prescribed NI are indicated by orange, as in Fig. 4. If the AvgPNI is above the Prescribed NI, it will be displayed in orange in the GSI widget as in Fig. 5.

**Figure 6:**
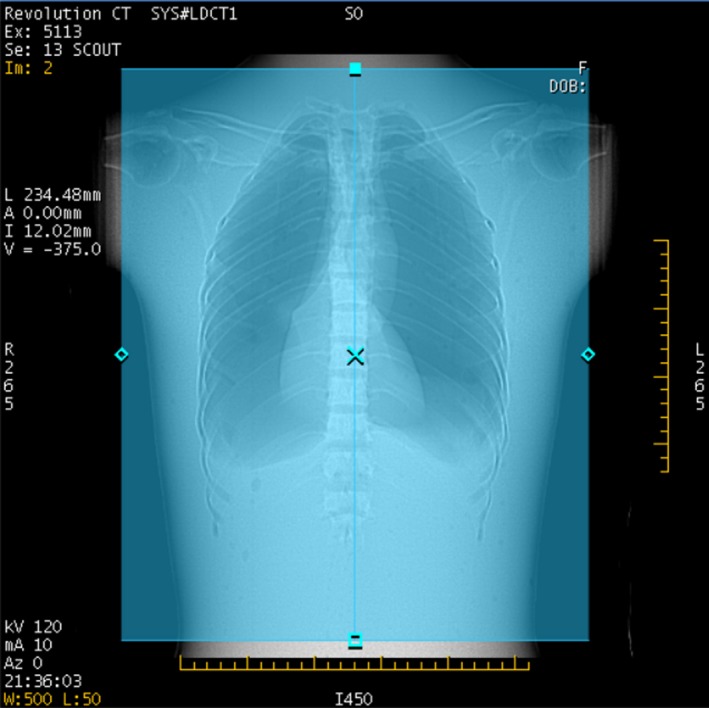
The scout view of the same scan after selecting the “Large Body/80 mm/L/1.0 s/0.508:1” scan settings. Because the projected noise is below the Prescribed NI for the entire scan, no orange sidebars are present.

**Figure 7:**
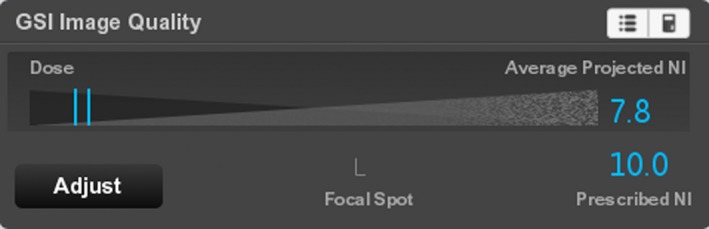
The GSI IQ widget for the same scan settings as Fig. 10. Since the Average Projected NI is below the Prescribed NI, it is displayed in blue. In this case, the radiation dose is higher than desired.

If the projected noise is below the Prescribed NI for the entire scan, no orange sidebars are displayed (Fig. 6). Whenever the Average Projected NI is equal to or below the target Noise Index, it will be displayed in blue (Fig. 7).

**Figure 8:**
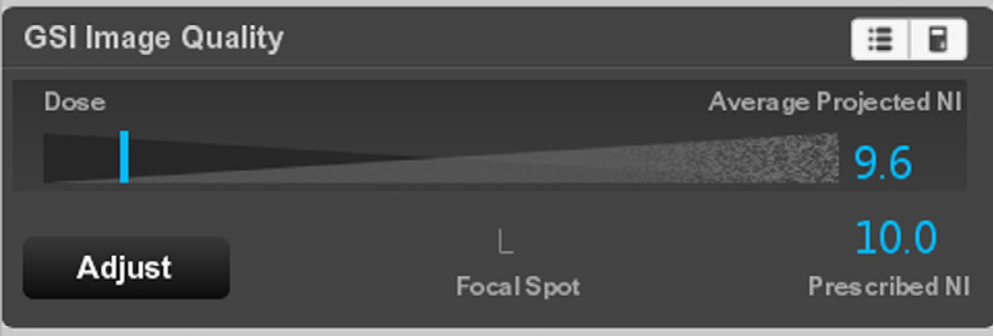
The GSI IQ widget for the same scan settings as Fig. 9.

**Figure 9:**
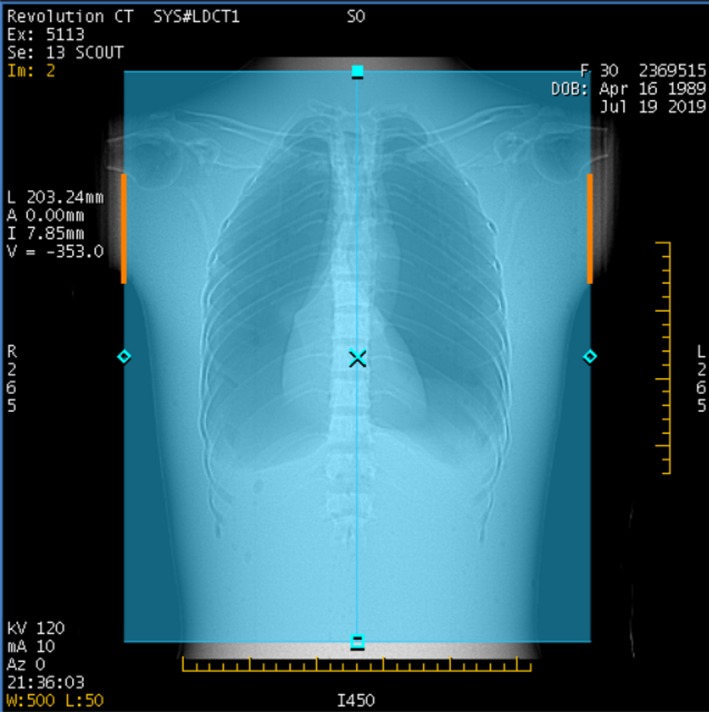
The scout view of the same scan after selecting the “Large Body/80 mm/L/0.8 s/0.992:1” scan settings. When the Average Projected NI is close to the Prescribed NI, orange sidebars are typically present for part of the scan.

As longitudinal tube current modulation is not used in GSI acquisitions, whenever the Average Projected NI is close to the Prescribed NI (Fig. 8), the projected noise is likely to be above the Prescribed NI for part of the scan. In this case, orange sidebars run along parts of the scout (Fig. 9).

**Figure 10:**
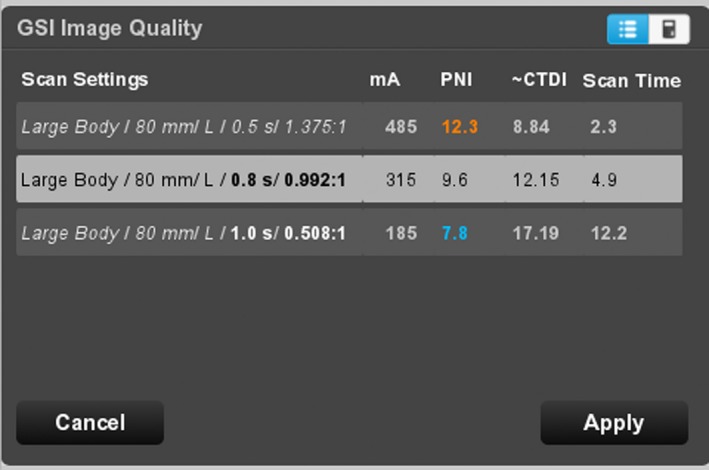
GSI Image Quality widget after clicking on the “Adjust” button (“Solutions window”). This display allows the technologist to change the Preset Family (“Scan Settings”). A yellow mA (not shown here) would indicate that the maximum tube current has been reduced to avoid tube overheating; an orange PNI indicates that the projected CTDI_vol_ is above the target CTDI_vol_.

The technologist has the option of changing the Preset Family selection by clicking on the “Adjust” button. The scanner will then display a Solutions dialog box (Fig. 10) listing each of the GSI Preset Families in the GSI Profile, as well as the projected noise index (PNI) and the CTDI_vol_ for the tube current selected for each Preset Family. The technologist may select any one of these Preset Families, overriding the present selection.

The number of options available in this dialog box can be substantial. There will be one row of data for each GSI Preset Family in the GSI Profile; for the GE Reference Profiles, this can be as few as three rows and as many as nine rows.

If the CID Link setting is turned on, the Clinical Identifier determines which GSI profile to use. If the CID Link setting for a GSI acquisition is turned off, the GSI Profile may be selected manually.

The goal of the method presented below is to design a set of GSI Profiles that

1. Allows the physicist to give the technologist simple, clear instructions on when to change the GSI Preset Family, and which GSI Preset Family to select, and
2. Makes available the widest possible range of CTDI_vol_ values, to ensure that the system does not produce excessively noisy images of large patients or use excessive radiation dose on small patients.

## 2 Method

At UAB Medical Center, we created User Profiles that contain only three GSI Preset Families per profile, one for small, one for medium, and one for large patients. The medium Preset Family is specified as the primary Preset Family.

For example, the Preset Families in the “UAB LIVER” profile are listed in Table 1, along with the minimum and maximum values of CTDI_vol_ that can be produced with each Preset Family. With these settings, the scanner can perform acquisitions that match the target CTDI_vol_ to within ±5% over most of the range 3.6 to 44.6 mGy.

**Table 1:** “UAB LIVER” GSI protocol.

Patient size	SFOV, focal spot, collimation	Pitch	Rot time (s)	Min CTDIvol (mGy)	Max CTDIvol (mGy)
Small	Large Body, Large, 80 mm	1.375	0.5	3.6	8.6
Medium (primary)	Large Body, Large, 80 mm	0.992	0.8	7.3	18.6
Large	Large Body, Large, 80 mm	0.508	1.0	17.4	44.6

Although the Preset Families in this table are listed in order of increasing CTDI_vol_, on the scanner the Medium Preset Family is listed first in order to identify it as the primary Preset Family.

We have instructed the technologists to use the primary Preset Family unless the PNI is more than 2 HU below or above the target noise index (the noise index represents the target noise in the CT number, so the noise index has units of HU). For a target noise index of 18, a difference of 2 HU in the noise index is 11%, which corresponds to a 22% mismatch in the CTDI_vol_. A change in the CTDI of less than ~25% does not result in a noticeable change in the noise,^9^ so this difference is not clinically significant.

If the PNI is more than 2 HU below the target noise index, the “Adjust” tool is used to select the “Small adult” Preset Family. If the PNI more than 2 HU above the target noise index, the “Adjust” tool is used to select the “Large Adult” preset.

### 2.1 Suggested approach to customizing GSI Protocols

The following approach can be used to customize GSI profiles to accommodate the needs of a specific facility for a specific type of exam. The goal of this procedure is to select three or four Preset Families whose CTDI_vol_ values overlap, but with as little overlap as possible. This allows a wide range of possible CTDI_vol_ values with few Preset Families, and thus simplifies the selection of Preset Family.

This procedure makes use of the tables in Appendix B. Each table in this Appendix describes Preset Families for a single combination of scan field of view and collimation. Each row of each table describes a single Preset Family. Each Preset Family includes multiple mA settings covering a range of values of CTDI_vol_, so there is a minimum and a maximum value of CTDI_vol_ listed. The rows are listed in order of increasing minimum CTDI_vol_.

1. Based on clinical considerations, determine the desired scan field of view, collimation, and the range of acceptable gantry rotation times and acceptable helical pitches.
2. Determine if use of the XLarge focal spot is acceptable; using this focal spot may reduce spatial resolution^9^ but allows higher tube currents and thus higher CTDI_vol_.
3. Find the table in Appendix B for the desired scan field of view and collimation.
4. Find the first Preset Family in the table that matches the restrictions on rotation time and helical pitch; this will be the “Small Patient” Preset Family.
5. Find the last Preset Family that
  - uses the large focal spot
  - matches the restrictions on rotation time and helical pitch, and
  - has a minimum CTDI_vol_ that is less than the maximum CTDI_vol_ of the “Small Patient” Preset Family.
  This will be the “Medium Patient” Preset Family.
6. Find the last Preset Family that
  - uses the large focal spot
  - matches the restrictions on rotation time and helical pitch, and
  - has a minimum CTDI_vol_ that is less than the maximum CTDI_vol_ of the “Medium Patient” Preset Family.
  This will be the “Large Patient” Preset Family.
7. Find the Preset Family with the highest CTDIvol that matches the restrictions on rotation time, helical pitch, and focal spot size. This will be the “Extra Large Patient” Preset Family. This may be the same as the “Large Patient” Preset Family; in this case the profile will have only three Preset Families.
8. Open the GSI Profile Editor, make a copy of an existing GE Reference Profile that contains the GSI Recons that you wish to use, and save it as a new User Profile.
9. Replace the Preset Families in the new User Profile with the Preset Families selected above. Place the “Medium Patient” Preset Family at the top of the list, identifying it as the primary Preset Family.
10. (Optional) Add or delete GSI Recons to the new User Profile as desired.

In selecting the Preset Families to include in a profile, consider the following^6^:

1. It has been observed in the GE Discovery scanners that material decomposition values are more accurate for low‐current, long rotation time combinations than at high‐current, short rotation time combinations.^10^ This is likely because for short rotation times, the time spent switching between tube voltages is a higher fraction of the total rotation time, reducing the accuracy of the acquired data. Therefore, use of longer rotation time allows more accurate material decomposition images at the expense of increased sensitivity to patient motion. This is more likely to be useful in body studies than in chest studies.
2. The use of the Extra Large focal spot allows higher tube currents, but may degrade spatial resolution.^11^ This is more likely to be a problem in MSK or chest protocols than in abdominal protocols.
3. As each Preset Family imposes a lower limit on the tube current, to achieve appropriately low radiation dose for smaller patients it may be necessary to include Preset Families with higher helical pitches. This is unlike single‐energy AEC systems, which will compensate for higher helical pitches by raising the tube current to maintain the same CTDI_vol_.^12^ However, the use of high helical pitch may introduce image artifacts.
4. The selection of the primary Preset Family may depend on your patient population. For example, if your facility images primarily bariatric patients, use the Large Patient Preset Family as the primary family. A pediatric hospital should use the Small Patient Preset Family as the primary family.
5. The range of available values of CTDI_vol_ will depend on the selected constraints. For example, allowing helical pitch values ranging from 0.508 to 1.531 will allow the use of both higher and lower values of CTDI_vol_ than available if the helical pitch is constrained to always be 0.992.

### 2.2 Example

Suppose that you wish to build a new GSI Profile based on the “GE Routine Abdomen Contrast” profile, using the large body field of view, 80 mm collimation, and helical pitches of no more than 1.375, and which may use the extra large focal spot.

1. See Table B1 in Appendix B; this lists the Preset Families for the large body SFOV and 80 mm collimation.
2. Find the first Preset Family with a helical pitch of no more than 1.375; the CTDI_vol_ for this Preset Family ranges from 3.4 to 8.8 mGy. This is the “Small Patient” Preset Family.
3. Find the last Preset Family that uses the large focal spot, has a helical pitch of no more than 1.375, and has a minimum CTDI_vol_ of less than 8.8 mGy; the CTDI_vol_ for this combination ranges from 7.1 to 18.9 mGy. This is the “Medium Patient” Preset Family.
4. Find the last Preset Family that uses the large focal spot with a helical pitch of no more than 1.375 and a minimum CTDI_vol_ of less than 18.9 mGy. The CTDI_vol_ ranges from 17.2 to 45.5 mGy. This is the “Large Patient” Preset Family.
5. Find the Preset Family with the highest maximum CTDI_vol_ and a helical pitch of no more than 1.375. The CTDI_vol_ ranges from 49.7 to 53.7 mGy. This is the “Extra Large Patient” Preset Family.

## 3 Results

**Figure 11:**
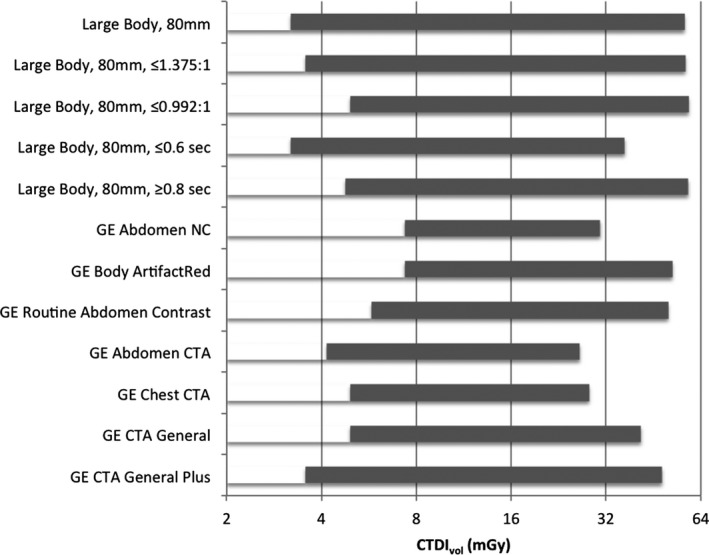
The range of CTDI_vol_ values that can be produced by various user GSI profiles produced with the procedure described in this paper, and by various profiles supplied by the vendor.

Fig. 11 presents the range of CTDI_vol_ values that can be produced with user GSI profiles that were generated using the procedure described in this paper, as well as vendor supplied abdomen and chest GSI profiles. The user GSI profiles generally allow the use both of higher CTDI_vol_ and lower CTDI_vol_ values than the vendor supplied profiles.

The user profiles shown in Fig. 11 are described in detail in Tables 2, 3, 4, 5, 6. These profiles are all constrained to use the large body scan field of view and an 80 mm detector coverage, but have various constraints on the helical pitch and on the rotation time. Note that in these tables, the Preset Families are all listed in order of increasing CTDI_vol._ When installing these on a scanner, the desired primary Preset Family should be placed at the top of the list.

**Table 2:** Large Body SFOV, 80 mm collimation.

Patient size	Preset Family (Scan FOV, focal spot, coverage)	Rot time (s)	Pitch	Min CTDI_vol_ (mGy)	Max CTDI_vol_ (mGy)
Small	Large Body, Large, 80 mm	0.5	1.531	3.1	7.9
Medium	Large Body, Large, 80 mm	1	1.375	6.3	16.8
Large	Large Body, Large, 80 mm	0.8	0.508	14.0	36.8
Extra large	Large Body, XLarge, 80 mm	1	0.508	49.7	53.7

This profile is designed for abdomen and pelvis protocols where patient motion is not an issue, so long rotation times and low pitches are acceptable.

**Table 3:** Large Body SFOV, 80 mm coverage, helical pitch ≤1.375.

Patient size	Preset Family (Scan FOV, focal spot, coverage)	Rot time (s)	Pitch	Min CTDI_vol_ (mGy)	Max CTDI_vol_ (mGy)
Small	Large Body, Large, 80 mm	0.5	1.375	3.4	8.8
Medium	Large Body, Large, 80 mm	0.8	0.992	7.1	18.9
Large	Large Body, Large, 80 mm	1	0.508	17.2	45.5
Extra large	Large Body, XLarge, 80 mm	1	0.508	49.7	53.7

This profile is designed for abdomen and pelvis protocols, where patient motion is not an issue but there is concern about artifacts from helical pitches larger than 1.375.

**Table 4:** Large Body SFOV, 80 mm coverage, helical pitch ≤ 0.992.

Patient size	Preset Family (Scan FOV, focal spot, coverage)	Rot time (s)	Pitch	Min CTDI_vol_ (mGy)	Max CTDI_vol_ (mGy)
Small	Large Body, Large, 80 mm	0.5	0.992	4.7	12.2
Medium	Large Body, Large, 80 mm	0.6	0.508	10.9	28.4
Large	Large Body, Large, 80 mm	1	0.508	17.2	45.5
Extra large	Large Body, XLarge, 80 mm	1	0.508	49.7	53.7

This profile is designed for abdomen and pelvis protocols, where patient motion is not an issue but there is concern about artifacts from helical pitches larger than 1.

**Table 5:** Large Body SFOV, 80 mm coverage, rotation time ≤0.6 s.

Patient size	Preset Family (Scan FOV, focal spot, coverage)	Rot time (s)	Pitch	Min CTDI_vol_ (mGy)	Max CTDI_vol_ (mGy)
Small	Large Body, Large, 80 mm	0.5	1.531	3.1	7.9
Medium	Large Body, Large, 80 mm	0.6	0.992	5.6	14.6
Large	Large Body, Large, 80 mm	0.6	0.508	10.9	28.4
Extra large	Large Body, XLarge, 80 mm	0.6	0.508	31.1	33.6

This profile is designed for chests protocols, where priority is given to fast scans in order to minimize motion artifacts.

**Table 6:** Large Body SFOV, 80 mm coverage, rotation time ≥0.8 s.

Patient size	Preset Family (Scan FOV, focal spot, coverage)	Rot time (s)	Pitch	Min CTDI_vol_ (mGy)	Max CTDI_vol_ (mGy)
Small	Large Body, Large, 80 mm	0.8	1.531	4.6	12.2
Medium	Large Body, Large, 80 mm	1	0.992	8.8	23.3
Large	Large Body, Large, 80 mm	1	0.508	17.2	45.5
Extra large	Large Body, XLarge, 80 mm	1	0.508	49.7	53.7

This profile is designed for abdomen protocols for which priority is given to quantitative pixel value accuracy.

Appendix A describes additional GSI user profiles for 40 mm detector coverage.

## 4 Discussion and Conclusion

The vendor supplied GSI profiles allow the scanner to match the PNI to the target noise index to within about 1%. However, the range of values of CTDI_vol_ available to the scanner when using some profiles is relatively limited, resulting in excess radiation dose for small patients and excess noise for large patients. In addition, to achieve the full precision of the PNI, the technologist must always select the right preset profile in the Image Quality Adjust tool. Thus it may be difficult in practice to achieve this 1% match in target noise index.

The user profiles presented here produce a wider range of CTID_vol_ values than the vendor supplied profiles at least in part by allowing a wider range of helical pitch and rotation time values. For example, neither of the vendor supplied profiles “GE Abdomen NC” and “GE Body ArtifactRed” use a helical pitch above 0.992, and so will never produce CTDI_vol_ values below 7.3 mGy, while the user profile “Large Body SFOV, 80 mm coverage” allows the use of a helical pitch of 1.531 with a rotation time of 0.5 s, allowing the use of acquisitions with CTDI_vol_ as low as 3.1 mGy.

By developing and using custom GSI Profiles using the technique described here, we have managed to both simplify the technologist’s task in selecting presets and increase the range of patient sizes that can be accommodated. However, these goals are achieved by allowing larger noise index error (up to 5%) than the reference GSI profiles for some imaging conditions. We believe that deviations at this level are clinically insignificant. Over the length of an abdomen/pelvis exam, the patient size may easily change by 5 cm; this corresponds to a change in patient transmission of about a factor of 2, or a 40% change in the image noise. An error of 5% in the average noise may be considered insignificant compared to the 40% variation in image noise along the length of the scan.

## Conflicts of Interest

No conflict of interest.

## APPENDIX A Additional GSI Profiles

The following tables describe GSI profiles for 40 mm detector coverage, and for the medium body field of view.

**Table A1:** Large Body SFOV, 40 mm coverage.

Patient size	Preset Family (Scan FOV, focal spot, coverage)	Rot time (s)	Pitch	Min CTDI_vol_ (mGy)	Max CTDI_vol_ (mGy)
Small	Large Body, Large, 40 mm	0.5	1.375	3.5	9.0
Medium	Large Body, Large, 40 mm	0.8	0.984	7.4	19.4
Large	Large Body, Large, 40 mm	1	0.516	17.3	45.7

**Table A2:** Large Body SFOV, 40 mm coverage, pitch ≤0.984.

Patient size	Preset Family (Scan FOV, focal spot, coverage)	Rot time (s)	Pitch	Min CTDI_vol_ (mGy)	Max CTDI_vol_ (mGy)
Small	Large Body, Large, 40 mm	0.5	0.984	4.9	12.6
Medium	Large Body, Large, 40 mm	0.6	0.516	11.0	28.6
Large	Large Body, Large, 40 mm	1	0.516	17.3	45.7

**Table A3:** Large Body SFOV, 40 mm coverage, rotation time ≤ 0.6 s.

Patient size	Preset Family (Scan FOV, focal spot, coverage)	Rot time (s)	Pitch	Min CTDI_vol_ (mGy)	Max CTDI_vol_ (mGy)
Small	Large Body, Large, 40 mm	0.5	1.375	3.5	9.0
Medium	Large Body, Large, 40 mm	0.6	0.984	5.8	15.0
Large	Large Body, Large, 40 mm	0.6	0.516	11.0	28.6

**Table A4:** Large Body SFOV, 40 mm coverage, rotation time ≥0.8 s.

Patient size	Preset Family (Scan FOV, focal spot, coverage)	Rot time (s)	Pitch	Min CTDI_vol_ (mGy)	Max CTDI_vol_ (mGy)
Small	Large Body, Large, 40 mm	0.8	1.375	5.3	13.9
Medium	Large Body, Large, 40 mm	1	0.984	9.1	24.0
Large	Large Body, Large, 40 mm	1	0.516	17.3	45.7

**Table A5:** Medium Body SFOV, 40 mm coverage.

Patient size	Preset Family (Scan FOV, focal spot, coverage)	Rot time (s)	Pitch	Min CTDI_vol_ (mGy)	Max CTDI_vol_ (mGy)
Small	Medium Body, Large, 40 mm	0.5	1.375	5.5	9.9
Medium	Medium Body, Large, 40 mm	0.8	1.375	8.4	15.1
Large	Medium Body, Large, 40 mm	0.8	0.984	11.8	21.1
Extra large	Medium Body, Large, 40 mm	1	0.516	27.6	49.6

**Table A6:** Medium Body SFOV, 40 mm coverage, pitch ≤0.984.

Patient size	Preset Family (Scan FOV, focal spot, coverage)	Rot time (s)	Pitch	Min CTDI_vol_ (mGy)	Max CTDI_vol_ (mGy)
Small	Medium Body, Large, 40 mm	0.5	0.984	7.8	13.8
Medium	Medium Body, Large, 40 mm	0.8	0.984	11.8	21.1
Large	Medium Body, Large, 40 mm	0.6	0.516	17.5	31.1
Extra large	Medium Body, Large, 40 mm	1	0.516	27.6	49.6

**Table A7:** Medium Body SFOV, 40 mm coverage, rot time ≤ 0.6 s.

Patient size	Preset Family (Scan FOV, focal spot, coverage)	Rot time (s)	Pitch	Min CTDI_vol_ (mGy)	Max CTDI_vol_ (mGy)
Small	Medium Body, Large, 40 mm	0.5	1.375	5.5	9.9
Medium	Medium Body, Large, 40 mm	0.6	0.984	9.2	16.3
Large	Medium Body, Large, 40 mm	0.5	0.516	14.8	26.3
Extra large	Medium Body, Large, 40 mm	0.6	0.516	17.5	31.1

**Table A8:** Medium Body SFOV, 40 mm coverage, rot time ≥ 0.8 s.

Patient size	Preset Family (Scan FOV, focal spot, coverage)	Rot time (s)	Pitch	Min CTDI_vol_ (mGy)	Max CTDI_vol_ (mGy)
Small	Medium Body, Large, 40 mm	0.8	1.375	8.4	15.1
Medium	Medium Body, Large, 40 mm	0.8	0.984	11.8	21.1
Large	Medium Body, Large, 40 mm	1	0.984	14.5	26.0
Extra large	Medium Body, Large, 40 mm	1	0.516	27.6	49.6

## APPENDIX B GSI Preset Families

These tables list the GSI Preset Families available on the Revolution (160 mm coverage version). Each table lists the combinations for a particular collimation (NxT) and scan field of view, and is sorted in increasing order of minimum CTDI_vol_.

This information is based on information in Table 11 of the Revolution CT User Manual, Chapter 12.^13^

**Table B1:** Preset families for 80 mm collimation, Large Body scan FOV.

Preset Family (Scan FOV, focal spot, coverage)	Rot time (s)	Pitch	Min CTDI_vol_ (mGy)	Max CTDI_vol_ (mGy)
Large Body, Large, 80 mm	0.5	1.531	3.1	7.9
Large Body, Large, 80 mm	0.5	1.375	3.4	8.8
Large Body, Large, 80 mm	0.6	1.531	3.6	9.4
Large Body, Large, 80 mm	0.6	1.375	4.0	10.5
Large Body, Large, 80 mm	0.8	1.531	4.6	12.2
Large Body, Large, 80 mm	0.5	0.992	4.7	12.2
Large Body, Large, 80 mm	0.8	1.375	5.2	13.6
Large Body, Large, 80 mm	0.6	0.992	5.6	14.6
Large Body, Large, 80 mm	1	1.531	5.7	15.1
Large Body, Large, 80 mm	1	1.375	6.3	16.8
Large Body, Large, 80 mm	0.8	0.992	7.1	18.9
Large Body, XLarge, 80 mm	0.5	1.531	8.7	9.4
Large Body, Large, 80 mm	1	0.992	8.8	23.3
Large Body, Large, 80 mm	0.5	0.508	9.2	23.9
Large Body, XLarge, 80 mm	0.5	1.375	9.7	10.5
Large Body, XLarge, 80 mm	0.6	1.531	10.3	11.2
Large Body, Large, 80 mm	0.6	0.508	10.9	28.4
Large Body, XLarge, 80 mm	0.6	1.375	11.5	12.4
Large Body, XLarge, 80 mm	0.8	1.531	13.4	14.4
Large Body, XLarge, 80 mm	0.5	0.992	13.4	14.5
Large Body, Large, 80 mm	0.8	0.508	14.0	36.8
Large Body, XLarge, 80 mm	0.8	1.375	14.9	16.1
Large Body, XLarge, 80 mm	0.6	0.992	15.9	17.2
Large Body, XLarge, 80 mm	1	1.531	16.5	17.8
Large Body, Large, 80 mm	1	0.508	17.2	45.5
Large Body, XLarge, 80 mm	1	1.375	18.3	19.9
Large Body, XLarge, 80 mm	0.8	0.992	20.6	22.3
Large Body, XLarge, 80 mm	1	0.992	25.4	27.5
Large Body, XLarge, 80 mm	0.5	0.508	26.1	28.3
Large Body, XLarge, 80 mm	0.6	0.508	31.1	33.6
Large Body, XLarge, 80 mm	0.8	0.508	40.3	43.5
Large Body, XLarge, 80 mm	1	0.508	49.7	53.7

**Table B2:** Preset Families for 40 mm collimation, Large Body scan FOV.

Preset Family (Scan FOV, focal spot, coverage)	Rot time (s)	Pitch	Min CTDI_vol_ (mGy)	Max CTDI_vol_ (mGy)
Large Body, Large, 40 mm	0.5	1.375	3.5	9.0
Large Body, Large, 40 mm	0.6	1.375	4.1	10.7
Large Body, Large, 40 mm	0.5	0.984	4.9	12.6
Large Body, Large, 40 mm	0.8	1.375	5.3	13.9
Large Body, Large, 40 mm	0.6	0.984	5.8	15.0
Large Body, Large, 40 mm	1	1.375	6.5	17.2
Large Body, Large, 40 mm	0.8	0.984	7.4	19.4
Large Body, Large, 40 mm	1	0.984	9.1	24.0
Large Body, Large, 40 mm	0.5	0.516	9.3	24.1
Large Body, Large, 40 mm	0.6	0.516	11.0	28.6
Large Body, Large, 40 mm	0.8	0.516	14.1	37.0
Large Body, Large, 40 mm	1	0.516	17.3	45.7

**Table B3:** Families for 40 mm collimation, Medium Body scan field of view.

Preset Family (Scan FOV, focal spot, coverage)	Rot time (s)	Pitch	Min CTDI_vol_ (mGy)	Max CTDI_vol_ (mGy)
Medium Body, Large, 40 mm	0.5	1.375	5.5	9.9
Medium Body, Large, 40 mm	0.6	1.375	6.6	11.7
Medium Body, Large, 40 mm	0.5	0.984	7.8	13.8
Medium Body, Large, 40 mm	0.8	1.375	8.4	15.1
Medium Body, Large, 40 mm	0.6	0.984	9.2	16.3
Medium Body, Large, 40 mm	1	1.375	10.4	18.6
Medium Body, Large, 40 mm	0.8	0.984	11.8	21.1
Medium Body, Large, 40 mm	1	0.984	14.5	26.0
Medium Body, Large, 40 mm	0.5	0.516	14.8	26.3
Medium Body, Large, 40 mm	0.6	0.516	17.5	31.1
Medium Body, Large, 40 mm	0.8	0.516	22.4	40.2
Medium Body, Large, 40 mm	1	0.516	27.6	49.6

## APPENDIX C Details of parameter sets

### GSI Preset Family

**Table C1:** List of the parameters specified by a Preset Family, and the values available for each parameter. Note that the Scan FOV specifies both the scan field of view and the bowtie filter used. The user cannot define new GSI Preset Families.

Parameter	Values
Scan FOV	Medium Body, Large Body
Rotation time	0.5, 0.6, 0.8, or 1.0 s
Detector coverage (collimation)	20 mm (Medium Large Body) or 40 mm (Large Body only)
Helical pitch	0.516, 0.984, 1.375 (20 mm collimation) or 0.508, 0.992, 1.375, 1.531 (40 mm collimation)
Focal spot	Large or Extra Large
Annotated mA	6 to 8 tube current values ranging from 185 to 570 mA

Parameters specified by a GSI Preset Family; this is a summary from Chapter 12, Section 6, Table 11 of the Revolution User’s Manual^13^. According to GE Medical Systems, Preset Families using the Adult Head SFOV will be added to a version of the software released late in 2018.

### GSI Recon

**Table C2:** List of the parameters included in a GSI Recon. The user can define custom GSI Recons. Note that other reconstruction options such as image thickness and interval are defined by the primary axial series from which the GSI Recon is built.

Parameter	Values
ASIR‐V	The iterative reconstruction parameter
GSI data file	Select “Yes” to be able to perform GSI Data File functions in the GSI viewer
GSI image type	Monoenergetic, Material decomposition, Virtual unenhanced, GSI High KVP, GSI KVP‐like
Recon type	The filter kernel
KeV	Used only by Monoenergetic image type. Determines the simulated photon energy
MAR	Select to “Yes” to use metal artifact reduction
Window level, window width	LUT parameters
MD pair	Used only by the Material Decomposition image type. Describes the two basis materials

Parameters specified by a GSI Recon.

### GSI Profile

**Table C3:** List of the parameters included in a GSI Profile. Any GSI acquisition needs to specify the GSI Profile to use. The user can define custom GSI Profiles.

Parameter	Values
Primary Preset Family	The GSI Preset Family that will be used unless the user overrides this choice
Description	Text description of the profile
GSI Recons	A list of the GSI Recon series that will be produced
GSI Preset Families	A list of the GSI Preset Families that the user can select using the “GSI Assist Tool”

Parameters specified by a GSI Profile.
